# The concept, importance and values of support during childbearing and breastfeeding – A discourse paper

**DOI:** 10.1002/nop2.1108

**Published:** 2021-11-06

**Authors:** Anette Ekström‐Bergström, Stina Thorstensson, Caroline Bäckström

**Affiliations:** ^1^ Department of Health Sciences University West Trollhättan Sweden; ^2^ Research Group Family Centered Health (FamCeH) University of Skövde Skövde Sweden; ^3^ School of Health Sciences University of Skövde Skövde Sweden

**Keywords:** birth, clinical practice, family, fathers, healthcare organizations, labour, mothers, partners, professional issues, theory

## Abstract

**Background:**

Professional support in childbearing has beneficial effects on childbirth experience, interactions within the family, breastfeeding and medical outcomes. However, more knowledge is needed about prerequisites for professional support to be valuable and satisfactory during childbearing.

**Aim:**

The aim of this discourse paper is to describe and explore prerequisites for professional support that are of value for women and their families during childbearing as well as how healthcare organizations can be formed to facilitate these prerequisites.

**Design:**

Discourse paper.

**Methods:**

This discourse paper is based on our own experiences and is supported by literature and theory.

**Results:**

Well‐functioning structures and processes facilitate professional support that leads to safe, secure, calm and prepared parents with the ability to handle the challenges of childbearing and parenting. When organizing care in childbearing, prerequisites for support needs must also be considered.

## 1 BACKGROUND

The value of professional support during childbearing has been explored in several studies, which have generated evidence that such support has beneficial effects on parental experiences, interactions between partners and with their baby and medical outcomes. However, like childbearing, the concept of support is complex, as will be discussed below.

### 1.1 The concept of support

Conceptually, support is described as an interactive process that can help individuals cope with challenging or stressful circumstances (Langford et al., 1997; Mander, 2008). Age, previous support experiences, and the social environment can affect one's ability both to provide and receive support (Langford et al., 1997). Perceptions of support are affected by the relationship between the provider and the recipient and are linked to attachment processes and social roles (Sarason et al., 1990). The perception of available support seems to be more important than the actual support itself (Uchino, 2003).

Support can be divided into *emotional, appraisal, informative* or *instrumental support*. *Emotional support* involves providing empathy, love and trust and promotes a sense of safety and belonging (Langford et al., 1997; Mander, 2008). *Appraisal support* involves help in self‐evaluation and promotes the reassurance of one's skills and competence. Emotional and appraisal support have been described as critical to the positive experience and impact of support (Langford et al., 1997; Mander, 2008), such as buffering the negative effects of stress (Cohen, 1992). *Informative support* involves offering information to help solve problems, whilst *instrumental support* (also called *practical support*) entails practical assistance for the same purpose (Langford et al., 1997; Mander, 2008). These different forms of support often overlap, for example, informative support may be accompanied by or perceived as emotional or appraisal support. Importantly, receiving support without asking for it seems to be vital for eliciting positive effects (Uchino, 2003) and thus proactive support is more beneficial than reactive support (Ericson et al., 2017; Thorstensson, et al., 2015; Thorstensson, et al., 2015). Asking for support is difficult for some, however, as it implies a sense of not being competent concerning the situation at hand (Uchino, 2003).

For professionals, providing support is a somewhat different approach than providing care. Support has been described as “letting someone lean on you,” whilst care involves “being responsible for” (Ernby, 2008; Hellqvist, 1980) another person. This suggests a difference in attitude as those who provide support must trust the capacity of the recipients, must approach them with caution and must let the recipients take the lead. In contrast, care involves more actively taking the lead and assuming responsibility for others (Thorstensson & Ekström, 2014).

The provision of support requires knowledge of universal needs (i.e. basic needs such as feeling safe and secure during childbirth) as well as the consideration of unique needs (some women feel safe at home, whilst others feel safe at hospital, during childbirth). In addition, a non‐judgmental attitude on the part of the provider is crucial for the recipient to experience support as positive (Hupcey & Morse, 1997; Langford et al., 1997; Oakley, 1994). However, it might be difficult for the provider to interpret the support needs of the recipient given situational complexities and ambiguities (Hertfelt Wahn et al., 2007). The provider may also hesitate to offer support for fear of inadvertently inflicting harm (Brownell & Schumacher, 1984) or due to stress (Hupcey & Morse, 1997).

Professional support in childbearing could be understood as “being with a woman,” which includes providing emotional and appraisal support (Bradfield, et al., 2019; Bradfield, et al., 2019). Learning to “be with a woman” has been described by midwifery students as requiring time and good role models (Kuliukas et al., 2020). Another concept close to professional support is watchful attendance, as described by de Jong et al. (2021), which addresses the “action” needed from the support provider during birth, for instance. This has also been described by Kennedy (2002) as “the art of doing nothing well.” Providing support during labour entails not just being with the woman (and her partner), providing support when needed or requested, but above all being attentive to her needs, which often change during the labour process. It is about *being with* rather than *doing things to* the woman (Bradfield, Hauck, et al., 2019; Bradfield, Kelly, et al., 2019).

### 1.2 Social and professional support

Support can be provided from one's own social network (such as family, friends, or significant others), i.e. *social support* or by professionals (such as midwives or nurses), i.e. *professional support*. Social support is reciprocal and builds on working relations that develop over time, whilst professional support is the job of health professionals and is, therefore, not a reciprocal, altruistic act. Professional support should aim to strengthen social support in order to strengthen the recipient (Hupcey & Morse, 1997).

Professional support aims to be empowering, which can be considered as both a process and an outcome (Oudshoorn, 2005). As a process, empowering support (i.e. empowerment) will strengthen individuals, and it is an important aspect of supportive midwifery or nursing care (Hermansson & Mårtensson, 2011). Empowerment can be described as a partnership in which professionals have power with the individual instead of having power over the individual (Labonte, 1994). This is in line with the notion that support demands a different attitude from caring, as it focuses on trusting the capacity of recipients and letting them “lead” the process (Thorstensson & Ekström, 2014). When professionals, as midwives and nurses, are empowering, they can have a beneficial impact on the lives and health of many individuals and families (Hermansson & Mårtensson, 2011).

### 1.3 Parents' need for support in childbearing

Childbearing is a major life‐changing event, especially for first‐time parents (Cowan, 2000). It is a transition that involves physical as well as psycho‐social challenges (Klobucar, 2016) with existential dimensions (Prinds et al., 2013). Expecting parents thus need substantial support in order to handle these challenges (Hildingsson & Thomas, 2007; Seefat‐van Teeffelen et al., 2011), and they should receive such support in various ways, i.e. in ways that address both universal and uniquely individual needs (Geary & Schumacher, 2012). Hence, in childbearing, support from midwives and nurses is important for interactions between expecting parents (Bäckström, 2018), for mother–infant interaction (Ekström & Nissen, 2006; Tarkka & Paunonen, 1996; Thorstensson et al., 2012), for breastfeeding (Ekström et al., 2006; Renfrew et al., 2012) and for the parents' overall childbirth experience (Bäckström & Hertfelt Wahn, 2011; Dahlen et al., 2010; Lundgren, 2004, 2005). Support that promotes a sense of security and calm can also have positive physiological effects in childbearing (Olza et al., 2020).

#### 1.3.1 Parents' need for support during pregnancy

Pregnancy is accompanied by physiological, psychological, as well as social contextual changes (Klobucar, 2016). Pregnancy actualizes existential reflections on the meaning of life (Prinds et al., 2013), which entails not only changing one's habits of mind and daily living (Klobucar, 2016) but also one's self‐image and relationships with social connections (such as the partner, family, and friends) (Geary & Schumacher, 2012; Meleis et al., 2000; Raphael‐Leff, 2005). Therefore, becoming pregnant for the first time means facing the unknown for both the expecting mother and her partner (Cowan, 2000), which includes handling uncertainty (Geary & Schumacher, 2012). Expecting parents' experiences with pregnancy vary, with some describing it as positive, strengthening and developmental, whilst others consider it negative or stressful (Buultjens et al., 2013). Pregnancy experiences may affect expecting parents' feelings of being prepared for childbirth and parenthood (Bäckström, 2018; Barimani et al., 2017; Ekström & Thorstensson, 2015). Further, these experiences may influence the transition to parenthood (Barimani et al., 2017; Schumacher & Meleis, 1994). The ways in which parents perceive and cope with life changes following childbearing may be connected with their family (“Sense of Coherence” [SOC]), since families with a high SOC may have better coping strategies (Antonovsky & Sourani, 1988; Bäckström et al., 2018). Nevertheless, support has been shown to facilitate the transition to parenthood (Barimani et al., 2017), and, therefore, expecting mothers need sufficient support to handle pregnancy‐related experiences and changes (Bäckström, Larsson, et al., 2017; Bäckström et al., 2016), whereas their partners need support in understanding the reality of pregnancy and how they can better support the expecting mother (Bäckström et al., 2020; Bäckström, Thorstensson, et al., 2017; Hrybanova et al., 2019; Huusko et al., 2018).

#### 1.3.2 Parents' need for support during labour and birth

During labour and birth, women face immense physical challenges, including pain, which demands them to focus and trust their body as well as the birthing process (Lundgren, 2004; Olza et al., 2018). Women thus require support to feel safe, secure and calm, to obtain and retain their inner strength – *integrative power*. If they do not receive such support, they might lose such qualities – *disintegrative power* (Fahy & Parratt, 2006; Nilsson, et al., 2012; Nilsson, et al., 2012), which could ultimately affect the birth process (Olza et al., 2020). Women's individual capacity to handle the challenges of labour and birth may vary, and thereby their needs during the birthing process will also change (Lundgren, 2004; Nilsson, Thorsell, Hertfelt Wahn, et al., 2012; Nilsson, Thorsell, Zandren Hammar, et al., 2012). Their partners, on the contrary, must address their own emotional reactions at the same time as they are assumed to be providing support. Partners' capacity to handle challenges during the birth process will fluctuate as well, as they may be subject to feelings of being left out, helplessness and, at the same time, excitement about the birth of their baby (Bäckström & Hertfelt Wahn, 2011; Ekström et al., 2013). If their partners feel helpless, expecting women may be unable to obtain or retain their inner strength, introducing disintegrative power into the relationship (Fahy & Parratt, 2006). The birth environment can also affect the expecting parents' feelings of safety and control during the birth process (Berg et al., 2012; Fahy & Parratt, 2006; Nilsson et al., 2020), as it may impact the ability of professionals to provide support since they feel more relaxed when they are allowed to “be with” the women (Davis & Homer, 2016).

#### 1.3.3 Parents' need for support during the first year after birth and breastfeeding

After the baby is born, mothers and their partners need to meet the baby feeling safe, secure and calm since such feelings facilitate the parental transition, parent–infant interactions, interactions between parents and breastfeeding (Barimani et al., 2017; Crenshaw, 2014). The parents and their baby also need skin‐to‐skin contact, which further strengthens parent–infant interactions as well as promotes breastfeeding and the well‐being of the mother and her baby (Crenshaw, 2014; Handlin et al., 2009; Widström et al., 2019). Skin‐to‐skin contact in the first hour after birth provides vital advantages to short‐ and long‐term health, the baby's temperature regulation and bonding (Widström et al., 2019). It also promotes instinctive behaviours on the part of the infant, maternal responses and attachment for up to 1 year after birth (Bystrova et al., 2009). As a consequence, the mother's confidence in her own and her baby's capability is facilitated, which in turn encourages the mother's transition to parenthood. Therefore, skin‐to‐skin contact between the baby and the mother is recommended in clinical practice (Widström et al., 2019), and should be included in professional support.

During the postnatal period, mothers need to process their birthing experiences and their new parenting role (Thorstensson, Andersson, et al., 2015), balancing feelings of happiness and the sense of being overwhelmed (Thorstensson, Hertfelt Wahn, et al., 2012). New fathers have the need to be acknowledged as a parent whilst at the same time understanding that the mother has her own needs. Fathers also want to meet other parents in, for example, parental groups (Hrybanova et al., 2019). Overall, new parents need information and knowledge of the baby's needs and care. First‐time mothers seek professional support about postnatal issues after birth because they often have difficulties preparing themselves for early parenthood during pregnancy. Hence, they need relevant information, encouragement in personal reflections and partner communication (Pålsson et al., 2017; Thorstensson, Andersson, et al., 2015). Expectant fathers also need support to develop strategies for the care of their baby and for the fatherhood role (Bäckström, Thorstensson, et al., 2017; Ekström & Thorstensson, 2015; Hrybanova et al., 2019). Professionals should, therefore, provide informative support based on parents' universal and individual needs.

Regardless of whether new mothers breastfeed their baby, they have unique support needs (Bäckström et al., 2010). However, breastfeeding includes physiological and psychological benefits for both the mother and the baby (Uvnäs Moberg et al., 2020), and as such parents need the requisite information for making informed choices concerning breastfeeding. When beginning breastfeeding, parents must have a calm and supportive environment, one which facilitates a sense of peace, self‐efficacy and security (Ekström & Thorstensson, 2015; Meedya et al., 2010). Positive experiences of breastfeeding by mothers generates the release of higher amounts of oxytocin, which, in turn, produces a sense of calm and security in mothers and enhances their interactions with their baby. Further, oxytocin promotes continued milk production (Uvnäs Moberg et al., 2020). First‐time mothers who enjoy breastfeeding, who have higher SOC, and who have more positive perceptions of the quality of their relationship with their partner seem to breastfeed for a longer time (Granberg et al., 2020).

#### 1.3.4 Continuity of care during childbearing

Expectant and new parents need to feel safe and secure during labour and birth, and continuity of care has been shown to facilitate this. Different types of continuity of care exist globally, examples of which are caseload midwifery and continuous labour support (one‐to‐one care). Caseload midwifery care means that the parental couple meets a team of midwives during pregnancy, labour and the postpartum period, building a continuous partnership (Kashani et al., 2021; Larsson et al., 2021). Continuous labour support means that women have a supportive companion who is continuously present and whose primary focus is the provision of support. This companion can be a midwife, a family relative or a doula. Women describe continuous support as enabling them to focus and endure their childbirth process (Lunda et al., 2018). Both midwifery students and newly graduated midwives are positive to work within kinds of healthcare organizations that enable them to form a relationship with women before birth (Cummings et al., 2015; Dawson et al., 2015), confirming support as an interactive process (Kahn & Antonucci, 1980). However, midwifery students also describe barriers to working within caseload midwifery, such as being on call and challenges to work–life balance and family commitments (Dawson et al., 2015). Midwife‐led continuity of care (such as caseload midwifery) has been shown to contribute to a higher rate of maternal satisfaction, according to a Cochrane review. This review also suggests that women who received midwifery‐led continuity of care are less likely to experience medical interventions during birth (Sandall et al., 2016). Also, continuous support helps women to handle childbirth pain (Van der Gucht & Lewis, 2015). As support is interactive, the provision of support will be strengthened when the provider focuses on one woman and her partner. Research has considered this to be especially important, as women's need for support during labour and birth might change (Lundgren, 2004) because the labour process is a powerful and dynamic physiological process (Olza et al., 2018). Hence, the aim of this discourse paper is to describe and explore the prerequisites of professional support in terms of their value for women and their families during childbearing as well as how healthcare organizations can be formed to enable this.

## 2 METHODS

This discourse paper is based on our own experiences and is supported by literature and theory.

## 3 RESULTS

**FIGURE 1:**
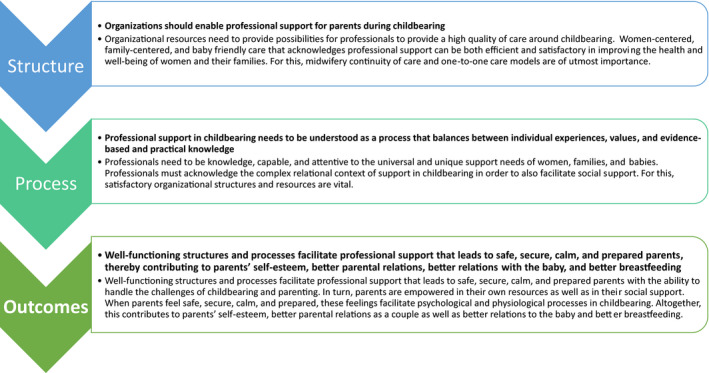
Prerequisites for professional support to be valuable and satisfactory during childbearing

Professional support in childbearing requires professionals to have knowledge about universal supportive needs. In addition, professionals must be attentive to the unique support needs of individual women, families and babies. In these regards, satisfactory healthcare organizational structures and resources are vital. Professionals must also acknowledge the complex relational context of support in childbearing in order to facilitate social support. With these prerequisites fulfilled, professionals can provide valuable and satisfactory support for women and their families during childbearing. An overview of the prerequisites is presented in Figure 1 and discussed below.

### 3.1 Structure: Healthcare organizations should enable professional support for parents during childbearing

Healthcare organizational resources should enable professionals to provide a high quality of care concerning childbearing. Woman‐ and family‐centred and baby‐friendly care that acknowledges professional support can efficiently and effectively promote the health and well‐being of women and their families. For this purpose, continuity of care by midwives and one‐to‐one care models comprise valuable cornerstones of utmost importance.

To encourage feelings of safety and calm among parents, continuity of care (such as midwife‐led continuity of care or caseload midwifery) (Sandall et al., 2016) and continuous labour support (also known as one‐to‐one care) (Bohren et al., 2017) have demonstrated beneficial effects (Bohren et al., 2017; Sandall et al., 2016). However, in most western countries, more women give birth in hospitals than at home or via midwife‐led continuity of care models. This means that continuous support during labour is often the exception rather than the norm, despite its beneficial effects (Bohren et al., 2017), such as a shorter active phase of labour and accompanying lower levels of the stress hormone cortisol compared to standard care (Stjernholm et al., 2021).

Continuous labour support has been shown, in repeated Cochrane reviews, to reduce the duration of labour and the number of medical interventions, increase the chance of spontaneous vaginal birth and positive childbirth experiences, and decrease the risk of babies having low 5‐min Apgar scores. There is no indication of harm being caused by the provision of continuous labour support (Bohren et al., 2017; Hodnett et al., 2011). In view of these facts, it is remarkable that the healthcare organization of care does not include continuous labour support in developed countries, such as Sweden – even more so given how many women mention missing support from midwives (Larsson et al., 2021; Lundgren, 2005), which can lead these women to feel neglected (Lunda et al., 2018). For their part, midwives consider the provision of professional support (i.e. emotional and appraisal support) to parents during childbearing to be one of their central roles. More specifically, as described by Davis and Homer (2016), these roles include promoting privacy, managing distractions and providing a calm and reassuring presence. Despite this, research has shown that labour ward workplace culture may prevent midwives from providing these functions of supportive labour care due to the healthcare organizational encouragement of “busy‐ness,” which demands that midwives care for more than one woman/couple at a time (Davis & Homer, 2016; Larsson et al., 2021). Hence, the healthcare organization of care during labour and birth may keep midwives from providing necessary types of support to the woman and her partner, despite their demonstrated beneficial impacts on both medical and psychological outcomes (Bohren et al., 2017). Another issue is that some midwives may be inattentive toward or neglect supportive needs, choosing to focus instead on more routine, standardized medical tasks during labour and birth. They may also lack sufficient training or competency in the provision of support during labour (Hunter, 2004; Thorstensson, Hertfelt Wahn, et al., 2012).

Midwifery students have described nursing training as task‐focused (Begley, 2001), which could mean being less prepared for providing the type of support that requires presence but not necessarily specific tasks to perform (Dahlen et al., 2010; Kennedy, 2002; Nilsson et al., 2010). However, when midwifery students are trained to offer continuous labour support to women and their partners, they are typically more aware of the importance of establishing relationships and rapport. They also become more cognizant of the impact of their presence on the birthing woman/couple. However, in learning situations, midwifery students often seek reassurance (i.e., appraisal support) regarding their capacity to provide support (Thorstensson et al., 2008). For students, reassurance from tutors (i.e. appraisal support) as well as approaching qualified midwives as role models in the provision of support to birthing women are integral (Zwedberg et al., 2020). If midwives are prevented from providing important dimensions of support due to healthcare organizational culture (Davis & Homer, 2016), midwifery students may not have sufficient opportunities to develop their supportive skills. Research has also shown that when midwifery students lack confidence in supportive situations, they tend to focus more on medical tasks than on their supportive role (Thorstensson et al., 2008). Midwifery students have described being with women (i.e. providing support) as making them more aware of the centrality of women in their care practices – a skill that took time to learn. Toward this end, positive role models, course workload as students and their student roles were all critical (Kuliukas et al., 2020; Zwedberg et al., 2020). The complexity of labour and birth together with the interactivity of support demand that midwifery education focuses on the duration and diversity of training in addition to the provision of good role models, i.e., midwives who provide continuous support during labour and birth.

Another type of professional support for parents is the provision of continuity of care from pregnancy to the baby's first year. This was studied in a longitudinal, randomized controlled trial in which midwives and child healthcare nurses in the intervention municipalities planned and performed parental group sessions from pregnancy to the end of the following year. The results demonstrated positive effects on perceived professional and social support, parent–infant feelings and relations, breastfeeding and partner participation (Ekström & Thorstensson, 2015). This is in line with other research, including interventions concerning extra professional support in which it was shown that extended continuity of care helps parents handle the new parental role, provide better care for their baby and establish more efficacious breastfeeding practices compared with traditional care (Abbass‐Dick & Dennis, 2018; Alberdi et al., 2018; Ekström & Thorstensson, 2015; Ericson et al., 2017; Nabulsi et al., 2919). Parents' need for support in their parenting and care of their baby could also be addressed by professional support offered through extended home visits.

Extended home visits for new parents strengthen parental knowledge, skills and motivation related to parenting and promote family health and function—an effect that can last through childhood and adolescence (Aronen & Arajarvi, 2000). Extended home visits also foster social support through social activities at child health centres as well as promote integration into, e.g. Swedish society for migrant parents (Bäckström et al., 2021). Previous research has reported that home visits provide early, individual and family‐centred support (Rautio, 2013), which strengthens both the self‐confidence and social networks of parents (Minkovitz et al., 2016). For instance, it has been found that parental confidence, knowledge of social services and local family resources can be strengthened for migrant fathers (Tiitinen Mekhail et al., 2019). In addition, home visits have been shown to reduce the incidence of child abuse (Hahn et al., 2003). This implies the importance of organizing care during childbearing in ways that will allow parents and their children to feel sufficiently safe and relaxed to express their support needs, which in turn helps professionals to recognize, understand and respond to such needs satisfactory.

### 3.2 Process: Professional support in childbearing needs to be understood as a process balancing between individual experiences, values and evidence‐based and practical knowledge

As previously mentioned, childbearing support is a complex phenomenon not only because childbearing is itself a complex process but also because the provision of support demands sensitive and knowledgeable interactivity. For childbearing support to succeed, professional, parental and social views must be considered and adequately balanced. But such balance can be affected by healthcare organizational structures and resources, as discussed above, as well as by the relative knowledge and skills of professionals. In the following, we will clarify and discuss professional and parental views on childbearing support, as well as its importance and value for parents.

Professionals, such as midwives, should possess appropriate and well‐developed skills but should also be given the working conditions needed to effectively balance between various supportive needs. When, during labour and birth, professionals are capable of providing women (and their partners) with emotional support (to feel safe), appraisal support (to feel able) and informative support (to feel knowledgeable), then such multifaceted support will allow women and their partners to negotiate and navigate the intense challenges associated with childbirth, such as uncertainty, pain or fear (Thorstensson et al., 2012). The support provider must, therefore, be attentive to the relative capacity of each woman to address these challenges, such as if the extent to which she can handle contraction decreases. That said, support providers must also be attentive to their own needs – otherwise, they may be incapable of meeting the needs of expectant mothers (Lundgren, 2004). Factors such as prolonged, unmitigated stress (Hupcey & Morse, 1997) and excessive workload may diminish midwives' ability to provide sufficient care (Berg et al., 2012). Women and their partners face a variety of complex challenges during pregnancy, labour and birth, as well as during the first year after birth. These include both physical challenges related to pregnancy and labour and emotional challenges like feelings of fear or exhaustion (Berg et al., 2012; Lundgren, 2004). For their part, partners often experience emotional stressors, such as the feeling of being left out, helpless or afraid, and they may also have difficulties understanding the reality of pregnancy and/or how to best support their partner (Bäckström & Hertfelt Wahn, 2011; Bäckström, Thorstensson, et al., 2017; Ekström et al., 2013). Clearly, childbearing entails existential issues that must be adequately acknowledged and addressed (Prinds et al., 2013). Here, we once again must remember that support during labour and birth is a complex, interactive task, one that must consider the well‐being of the woman, her partner and their baby, as well as the midwife and other professionals. Professionals, such as midwives, should possess the requisite skills and be provided with acceptable working conditions to effectively balance all of the supportive needs of the expecting parents so as to facilitate beneficial medical and emotional outcomes. Overall, it is important that both the woman and her partner feel safe and calm since these feelings will allow them to better handle childbearing and associated parental challenges. With adequate support (such as emotional and appraisal support), women can both obtain and retain their inner strength and their partners can support them in these regards – integrated power (Fahy & Parratt, 2006). Ultimately, such support will ensure the beneficial physiological outcomes of childbearing (Olza et al., 2020).

### 3.3 Outcomes: Well‐functioning structures and processes facilitate professional support that leads to safe, secure, calm and prepared parents, in turn contributing to parents' self‐esteem, better parental relations, better relations to the baby and better breastfeeding

Well‐functioning structures and processes within healthcare organizations facilitate professional support that leads to safe, secure, calm and prepared parents with the capacity to handle the challenges of childbearing and parenting. More precisely, such parents will be empowered in terms of their own resources and access to social support, as well as the availability of requisite psychological, physiological and social processes. Altogether, these factors contribute to enhancing parents' self‐esteem, improving their parental relations as a couple, strengthening their relation to their baby and encouraging healthy breastfeeding practices.

Nevertheless, the quality of the healthcare organizational structures associated with professional support for parents varies. When comparing professional support provided in small and large groups of expecting parents, research has shown no difference in their effects on first‐time mothers' childbirth experiences, parental skills (Fabian et al., 2005), stress levels or parenting relations 6 months after birth (Catling et al., 2015; Koushede et al., 2017). However, professional support provided in small groups has been demonstrated to broaden parents' social network and thereby increase the availability of social support (Bäckström et al., 2016; Bäckström, Thorstensson, et al., 2017; Barimani et al., 2017; Ekström & Thorstensson, 2015; Fabian et al., 2005). A randomized controlled trial showed that a combination of large‐group antenatal classes, “inspirational lectures,” and small groups of expecting parents seemed to increase the perceived quality of the parental relationship, e.g., consensus and sexuality, as well as its manageability after birth, in the eyes of parents (Thorstensson et al., 2020). Higher perceived social support during pregnancy has also been shown to enhance the perceived quality of parental relationships 6 months after birth (Bäckström et al., 2018), which implies that professional support during pregnancy must be provided in various ways in order to meet both universal and unique needs during the dynamic parental transition.

It is important to understand that the support during labour and birth also includes the provision of such support, e.g. appraisal and informative support, to the partner as well. Unsupported partners' capacity to support the expectant mother could diminish due to emotions such as helplessness or fear (Bäckström & Hertfelt Wahn, 2011; Ekström et al., 2013). This could in turn cause the expecting mother to lose focus, damage her sense of calm and feeling of security and compromise her ability to obtain and retain inner strength (Fahy & Parratt, 2006), which could ultimately disturb the physiology of birth (Olza et al., 2020). When women are supported to believe in their ability to give birth, and when the physiology of birth is not disturbed, the birth experience can be empowering despite being intensely challenging (Olza et al., 2018). This could be the reason why continuous labour support has been shown to have such beneficial effects (Bohren et al., 2017). Continuous support has also been demonstrated to lead to feelings of joy, pride and empowerment among new mothers, better preparing them to meet the varied and substantial needs of their newborn baby (Olza et al., 2018).

The value of professional support during pregnancy has been explored in several studies, and there is evidence of its profound effects on medical outcomes, such as a lower number of pre‐term births (Ickovics et al., 2007), reduced perinatal mortality (Dowswell et al., 2015), decreased fear of birth among first‐time mothers (Kacperczyk‐Bartnik et al., 2019) and reduced rates of caesarean births (Cantone et al., 2018). Besides, professional support seems to increase parental knowledge and feelings of being prepared for childbirth (Bäckström et al., 2016; Bäckström, Thorstensson, et al., 2017; Barimani et al., 2017; Gagnon & Sandall, 2007; Huusko et al., 2018; Svensson et al., 2009) and parenthood (Bäckström et al., 2016; Bäckström, Thorstensson, et al., 2017; Barimani et al., 2017; Ekström & Thorstensson, 2015; Manant & Dodgson, 2011; Svensson et al., 2009; Tiitinen et al., 2014). Professional support also facilitates a better relationship between the parental couple, with increased feelings of togetherness and improved communication skills (Bäckström et al., 2016; Bäckström, Thorstensson, et al., 2017).

For professionals to be able to provide sensitive professional support, they must naturally be attentive to parents' support needs (Hunter, 2004; Thorstensson, Ekström, et al., 2012). In order to improve their capacity to provide support, professionals have attended process‐oriented training in antenatal care and child health centres (Ekström & Thorstensson, 2015). This training was shown to promote attitudes towards parental support and breastfeeding among health professionals that were more facilitative and less regulating. This in turn led to mothers in the intervention group experiencing improved self‐reported relations to and feelings for the baby, regardless of whether the birth was normal or caesarean (Thorstensson, Andersson, et al., 2015; Thorstensson, Nilsson, et al., 2015; Thorstensson et al., 2012). As well as improved preparation for the parental role, breastfeeding became more prevalent and partners became more actively supportive, via parental support groups. In addition, the intervention group mothers also reported more positive perceptions of professional support from staff at the labour and maternity ward than mothers from the control groups (Ekström & Thorstensson, 2015), despite the fact that these professionals were not included in the process‐oriented training program. This suggests that improved support during pregnancy strengthens the woman, which may reduce her need for professional support whilst simultaneously increasing her ability to experience the support she does receive as positive. Earlier experiences of support have a bearing on current support experiences (Hupcey, 1998; Kahn & Antonucci, 1980), with a positive history of past support making expectant mothers more amenable to present support (Hupcey, 1998; Uvnas‐Moberg et al., 2005). Longitudinally speaking, we can only speculate on how improved professional support may influence the well‐being of mothers, their partners, and their families in the long run. This is an important consideration, however, since research has also shown that when women experience a lack of support during the first weeks after birth, emergency visits may increase due to breastfeeding concerns or fears over the health of the baby or mother herself (Barimani et al., 2014).

After their baby is born, parents often describe experiencing great happiness whilst simultaneously being overwhelmed by their responsibility for their baby (Thorstensson, Hertfelt Wahn, et al., 2012). Therefore, it is of great importance for such feelings to be acknowledged and attended to by health professionals and that practical tasks are not prioritized instead; these tasks, such as weighing the child, can usually wait until the parents and baby are ready.

To conclude, many years of research have demonstrated the importance of support and midwifery models of care. Therefore, we, as Renfrew et al. (2019) contended, assert that the global community should act in accordance with all available evidence concerning the benefits of midwifery care for women and their families. This is important to achieve sustainable health in line with the 2030 Agenda goals (Programme, 2019), in which midwives have been acknowledged as a critical resource in the promotion of high‐quality care for parents and newborns (Kemp et al., 2021).

## 4 CONCLUSION

For parents, childbearing is, clearly, mostly both a happy and challenging process within a complex relational context, and the ongoing interactivity includes both the parents' social network and healthcare professionals. Expecting parents need support in various ways during pregnancy, labour, and birth, as well as in the first year after birth and beyond since their needs are constantly changing during the dynamic childbearing and parental transition processes. When expecting parents' support needs are met, their ability to handle challenges and adapt to the parenting role will increase, and basic physiological processes will be facilitated. For professionals, it is important to have this knowledge because professional support should also strengthen social support. When organizing care in childbearing, prerequisites for support needs must be considered.

## 5 IMPLICATIONS FOR NURSING AND MIDWIFERY HEALTHCARE ORGANIZATIONS

- When organizing care in childbearing, parents' needs for support should be considered.
- Healthcare organizations should enable professionals to provide various types of professional support based on parents' universal and unique individual needs and requests. For this, various types of care healthcare organizations are needed (i.e., caseload midwifery or continuity of care) as well as numerous kinds of professional support (i.e., continuous support).
- Healthcare organizations should provide professionals with education and training that improve their knowledge of parents' needs and enhance the skills needed to meet these needs in complex contextual circumstances. Providing support means trusting the capacity of the recipients and creating possibilities for their individual development and growth within the context of their own unique needs and circumstances.

## CONFLICT OF INTEREST

The authors have no conflict of interest to declare.

## AUTHOR CONTRIBUTIONS

All authors have contributed equally to the ideas and formulation of this discourse article.
